# Volatiles from Subtropical Convolvulaceae That Interfere with Bacterial Cell-to-Cell Communication as Potential Antipathogenic Drugs

**DOI:** 10.1155/2016/7890260

**Published:** 2016-05-22

**Authors:** María C. Luciardi, María V. Pérez Hernández, Nora Muruaga, Alicia Bardón, Mario E. Arena, Elena Cartagena

**Affiliations:** ^1^Instituto de Química Orgánica, Facultad de Bioquímica, Química y Farmacia, Universidad Nacional de Tucumán, Ayacucho 471, 4000 Tucumán, Argentina; ^2^Fundación Miguel Lillo, Miguel Lillo 251, 4000 Tucumán, Argentina; ^3^INQUINOA-CONICET, Ayacucho 471, 4000 Tucumán, Argentina

## Abstract

Increasing chronic bacterial infections create an urgent need for new antimicrobial agents or strategies for their control. Targeting virulence is one of the alternative approaches to find new medicines to treat persistent infections due to bacteria with biofilm-phenotype which are more resistant to antibiotics than their planktonic counterparts having an extreme capacity for evading the host defences. A bioguided study of sixteen extracts from flowers and leaves of four subtropical Convolvulaceae species provided evidence of the occurrence of antipathogenic natural products active against Gram positive and negative bacteria. Particularly, volatile metabolites from* Merremia dissecta* creeper, a food and medicinal plant, were able to interfere with the* Pseudomonas aeruginosa* quorum sensing system by a strong decrease of* N*-acyl homoserine lactone (AHL) biosynthesis (63–75%), which attenuated the virulence factor expression like biofilm (55%) and elastase activity (up to 27%), key factors that enable the colonization and dissemination of the infection in the host. Control of the* P. aeruginosa* biofilm and the QS process by phytochemicals, such as (+) spathulenol, isolated from a bioactive extract of* M. dissecta* leaves would be a good strategy for the development of new and effective antipathogenic drugs.

## 1. Introduction

Microbes like bacteria can coordinate gene expression as a community through the secretion and detection of signalling molecules so that its members can simultaneously express specific behaviors. This mechanism of regulation, called quorum sensing (QS), seems to be a key trait for adaptation to specific environments and has been shown to regulate a variety of important phenotypes in the production of virulence factors like biofilm and proteolytic enzymes, crucial factors that enable colonization and dissemination of the infection in the host. Persistent and chronic bacterial infections have been directly linked to the presence of microbial biofilms. Microbial biofilms are sessile communities of one or more microorganisms that reside within a self-produced extracellular matrix. These cellular aggregates can form on living and nonliving surfaces, soluble and insoluble materials, are fairly ubiquitous in natural ecosystems, and have serious implications to human health. The inherent characteristic of microbial biofilms is a remarkable tolerance to treatment with antibiotics traditionally effective against planktonic (free floating) bacteria [1–4].

Biofilm infections, such as pneumonia in cystic fibrosis patients, chronic wounds, chronic otitis media, and implant and catheter associated infections, affect millions of people in the developed world each year and many deaths occur as a consequence [5].

There is evidence that bacterial QS process is involved in cross-kingdom signalling with eukaryotic organisms, mainly plants, that are capable of producing compounds that can interfere with QS systems in bacteria by inhibiting QS signal biosynthesis, which leads to attenuation of virulence factor expression [6]. Our investigations take inspiration from nature's models and attempts to design solutions to the problem of microbial biofilms [7–11].

Medicinal plants have become part of complementary medicine worldwide, because of their potential health benefits. Various plant extract has great potential against infectious agents and can be used for therapeutic purposes [12].

Convolvulaceae is a large family comprising about 58 genera and 1650 species of herbs, climbers, or shrubs distributed in tropical and subtropical regions [13]. The greatest species diversity occurs in the Americas and Africa. The family can be recognized by their funnel-shaped radially symmetrical corollas. The leaves and tuberous roots of some species are used as foodstuffs, and the seeds are exploited for their medicinal value as purgatives. Some species contain ergoline alkaloids that are likely responsible for the use of these species as ingredients in psychedelic drugs. The presence of ergolines in some species of this family is due to infection by fungi related to the ergot fungi of the genus* Claviceps*. The morning glory family is a rich source of bioactive compounds such as polyketides, terpenoids, steroids, flavonoids, coumarins, alkaloids, cyanogenic glycosides, and other compounds [14–16].

Several antibacterial activity studies of Convolvulaceae species and only a few about antibiofilms effects were previously published [17–22]. Therefore, this investigation presents a bioguided study of* Ipomoea cairica*,* I. nil*,* I. purpurea*, and* Merremia dissecta* that led to the discovery of plant volatiles that interfere with the* Pseudomonas aeruginosa* QS system, by declining of the QS signal production which attenuates virulence factor expression like biofilms and elastase* B* activity. In vitro antibiofilm effects against* Staphylococcus aureus* are also reported for the first time in this paper.

## 2. Materials and Methods

### 2.1. Collection and Identification of Plant Materials

 Leaves and flowers of* Ipomoea cairica* (L.) Sweet,* I. nil* (L.) Roth,* I. purpurea* (L.) Roth, and* Merremia dissecta* (Jacq.) Hallier f. (Convolvulaceae) were collected during the flowering stage in* March, road to Villa Nougués* (26°51′28.08′′S, 65°22′32.16′′W), Tucumán province, Argentina. Their identities were confirmed by Dr. Nora Muruaga and voucher specimens (LIL 610.873, 610.871, 610.870, and 610.872, resp.) were deposited at the Herbarium of Fundación Miguel Lillo, Tucumán, Argentina.

### 2.2. Preparation of Plant Extracts

The fresh plant material (leaves or flowers, 25 g) was extracted at room temperature for 3 days with small volume of diethyl ether (twice). The extracts from flowers and leaves were dried in rotary evaporator (Büchi R-3000) under vacuum at 30°C to give the following extracts: diethyl ether extracts from flowers (DEF) and leaves (DEL) and methanol extracts from flowers (MEF) and leaves (MEL).

### 2.3. Ultraviolet-Visible (UV-Vis) Analysis

UV-Vis spectra were measured in ethanol using a Shimadzu UV-Vis 160 A spectrophotometer in the range 200–800 nm to detect chromophoric systems with pharmacological potential.

### 2.4. Preliminary Screening to Select a Promising Bioactive Extract

#### 2.4.1. Effects of Plant Extracts on Bacterial Growth

Overnight cultures of* P. aeruginosa* ATCC 27853 and* Staphylococcus aureus* ATCC 6538 P were diluted to reach 2.5 × 10^6^ CFU/mL in Luria-Bertani (LB) and Mueller Hinton medium, respectively. The diluted culture (190 *μ*L) was placed in each of the 96 wells of a microtitre polystyrene plate. Solutions containing 0.1 mg/mL of extracts in DMSO–H_2_O (50 : 50) were prepared separately and 10 *μ*L of each was pipetted to the plastic microtitre plate wells individually (8 replicates). Control wells (8 replicates) contained the diluted culture (190 *μ*L) and 10 *μ*L of a solution of DMSO–H_2_O (50 : 50) in which the final concentration of DMSO was 2.5%. Medium control was prepared using sterile LB (or MH). Bacteria were cultured in LB (or MH) medium at 37°C and growth was detected as turbidity (600 nm or 560 nm) using a microtitre plate reader (Power Wave XS2, Biotek, VT, USA) and by direct counting of CFU/mL determined by plating 0.1 mL of the inoculation onto LB agar (pH 6.0). The maximum level of DMSO to which the cells were exposed was 2.5%.

#### 2.4.2. Antibiofilm Effects

For biofilm quantification, a micromethod based on a protocol previously reported was employed [23]. Biofilms formed after 24 h incubation of bacterial cultures prepared as described in the previous paragraph were stained with 20 *μ*L of an aqueous solution of crystal violet (0.1%, w/v) for 20 min. After washing with water, the liquid was discarded from the wells and the material that remained fixed to the polystyrene (containing biofilm) was washed with PBS (thrice). Crystal violet bound to biofilm was removed from each well employing 200 *μ*L absolute ethanol during 30 min at 37°C with shaking. Absorbance (540 nm) of ethanol solutions of crystal violet was determined using a microtitre plate reader (Power Wave XS2, Biotek, VT, USA). Azithromycin (5 *μ*g/mL), a known quorum sensing inhibitor, was incorporated in the bioassay as a control in the same experimental conditions employed to evaluate the compounds [24].

### 2.5. GC-EIMS and Isolation of Main Compound from a Bioactive Extract

On the basis of antibacterial and antibiofilm properties an extract among sixteen was selected and analyzed by gas chromatography techniques. GC-EIMS was carried out using a Thermo Electron Trace*™* Ultra couple with split-split less injector and Polaris Q instrument (Thermo Scientific, TX, USA) ion trap mass spectrometer equipped with a DB-5 capillary column (30 m × 0.25 mm, film thickness 0.25 *μ*m). The initial temperature of the column was 60°C during 0 min. A temperature programming was applied from 60°C to 246°C at a rate flow of 3°C/min and finally 280°C for 15 min. Carrier gas was helium (flow 1 mL/min). Split injection mode is 1/10. The identification of volatile components was based on computer matching with the NIST08 GC/MS library and by comparison of the mass spectra, retention times (RT), and Kovats retention indexes (RI) with those reported in the literature [25].

### 2.6. Chromatography Techniques

Diethyl ether extract from leaves of* M. dissecta* (DEL, 1 g) were fractionated on silica gel (70–230 Mesh, 1 : 50 w/w) column chromatography employing petroleum ether and increasing amounts of EtOAc (0–100%) and finally MeOH as mobile phase. The fourteen fractions were monitored by TLC on aluminium-precoated silica gel plates (Merck, Kieselgel 60 F_254_). The spots on the plates were visualised under UV light, and the plates were then sprayed with Godin reagent.

Fraction 4 isolated from DEL with a yield of 7.18% (according to Section 3) was submitted to GC-EIMS analysis and the main volatile compounds were determined. Then, this fraction was rechromatographed with more silica gel ratio (1 : 100, w/w) to give a known sesquiterpene whose NMR and EIMS spectra were measured.

### 2.7. Antibacterial and Antibiofilm Activities of* M. dissecta* Fractions

The bacterium screened was a biofilm phenotypic variant of* Pseudomonas aeruginosa* ATCC 27853, and the medium and techniques employed were previously described.

### 2.8. Antielastase *β* Activity of* M. dissecta* Fractions

Elastolytic activity was determined using a modification of the method previously described [26]. The substrate of the enzyme *β*-elastase, elastin Congo red (100 *μ*L) (Sigma), was dissolved in Tris-HCl (pH 8.0) at a concentration of 5 mg/mL and then it was mixed with 100 *μ*L of cell-free culture supernatant obtained from* P. aeruginosa* ATCC 27853 grown during 24 h, in LB media containing 5 *μ*g/mL of fractions, respectively. The reaction mixture (200 *μ*L) was incubated at 37°C for 24 h and centrifuged at 13,000 rpm for 10 min. The absorbance (495 nm) of the supernatant is a measure of the enzyme activity.

### 2.9. Autoinducer (AHL) Quantification

The interruption of bacterial cell-to-cell communication was deduced by autoinducer (AHL) quantification, using *β*-galactosidase activity assay.* P. aeruginosa* qsc 119 (reporter strain) is a mutant donated by Dr. Greenberg that cannot produce its own AHL (QS signal molecules). The reporter strain responds, by producing *β*-galactosidase, to exogenous active signal molecules generated by wild-type* P. aeruginosa* strains.* P. aeruginosa* qsc 119 was constructed using a chromosomal promoter under the control of AHLs linked to lacZ. In consequence, *β*-galactosidase activity is under QS-control and in direct relationship with the AHL activity [27].

An overnight culture of the reporter strain grown at 37°C in LB was diluted ten times in the same medium, reaching values of absorbance of 0.26 at OD_560 nm_. A 100 *μ*L portion of this suspension was mixed, in each microplate well, with 100 *μ*L cell-free culture supernatant obtained from* P. aeruginosa* ATCC 27853 cultured in LB media containing 100 *μ*g/mL of fractions, during 24 h. Azithromycin, known to interfere with the QS process, was used at 5 *μ*g/mL, concentration unable to affect the bacterial growth, as QS positive control under the same conditions as fractions [23]. Control wells (8 replicates) contained cell-free culture supernatant (100 *μ*L) obtained from* P. aeruginosa* ATCC 27853 cultured in LB media (190*μ*L) plus 10 *μ*L of DMSO–H_2_O (50 : 50). *β*-galactosidase activity was measured spectrophotometrically by Miller test [28].

### 2.10. Statistical Analysis

Differences between means were evaluated by analysis of variance (ANOVA). The Tukey test was used for all pair-wise multiple comparisons of groups. In all statistical analysis *P* values > 0.05 were not considered significant. Statistix 10 data analysis software for researches (2013) was used.

## 3. Results and Discussion

### 3.1. UV Absorptions and Yields of Convolvulaceae Extracts

All extracts showed a strong UV absorption (298–380 nm), a typical feature of the occurrence of aromatic compounds. These results are in agreement with the Convolvulaceae chemistry as it has already mentioned, since these metabolites are well known for their biological and pharmacological potential. It is important to note that the MeOH extracts (ME) exhibited the highest extraction yields (1.43–3.11%) reaching 3.11% in the ME of* I. nil* leaves (Table 1).

### 3.2. Antibacterial and Antibiofilm Activities of Convolvulaceae Extracts

The diethyl ether extracts (DE) were more active than methanol ones against* P. aeruginosa* growth as shown in Table 1. The antibacterial activity could be due to the presence of lipophilic compounds with lower polarity than cholesterol (according to TLC profile). This fact suggests that their principal targets are cell membranes and their toxicity would be caused by loss of chemiosmotic control as previously reported [29]. In relation to cell growth of* S. aureus*, the in vitro results demonstrated that both extracts are slightly active (Table 1).

Nevertheless, the addition of small amounts of MeOH extracts into* P. aeruginosa* cultures produced a lower biofilm-biomass than those of EE, except for* M. dissecta* DEL (diethyl ether extract from leaves) which only allowed a 20% biofilm formation (Table 1). In addition, this effect was correlated with an important inhibition of bacterial growth.* S. aureus* biofilm-biomass was also strongly reduced by all extracts (71–100%), and these effects were not growth dependent.* M. dissecta* MEF (MeOH extract from flowers) gave the best biofilm inhibition (100%).

### 3.3. Selection of a Promising Extract for Bacterial Biofilm Control

*M. dissecta* DEL, which reduced* P. aeruginosa* biofilm by 80%, was selected among sixteen extracts to continue studies. As it is unusual for natural products to be more active against Gram negative than positive bacteria [30], it is very important to find active compounds against pathogenic* P. aeruginosa*, which is a major cause of infection in immune-compromised patients. Indeed, these bacteria can cause serious infections in patients who have received massive antibiotic therapy, suffered severe burns, contracted HIV, or have a genetic disease like cystic fibrosis [31].

*M. dissecta* has been employed traditionally as a condiment, medicine, and ornament by an array of cultures. In Argentina, roots of* M. dissecta* var.* edentata* are still used as food by a few indigenous groups [16].

### 3.4. GC-EIMS Profile of* M. dissecta* Diethyl Ether Extract

GC-EIMS analysis of* M. dissecta* DEL has led to the identification of nine plant volatile compounds (Table 2) such as germacrene D (25.56%), *β*-caryophyllene (13.47%), spathulenol (6.27%), *β*-elemene (4.70%), and *δ*-elemene (2.65%) (sesquiterpenes), while benzyl alcohol (11.17%), benzoic acid (3.36%), and long-chain saturated fatty acids (17.28%) were also determined. Thus,* M. dissecta* is a promising source of sesquiterpenes and aromatic compounds with important bioactivities.

### 3.5. Fractionation of* M. dissecta* Diethyl Ether Extract and Bioactivities

**Figure 1:**
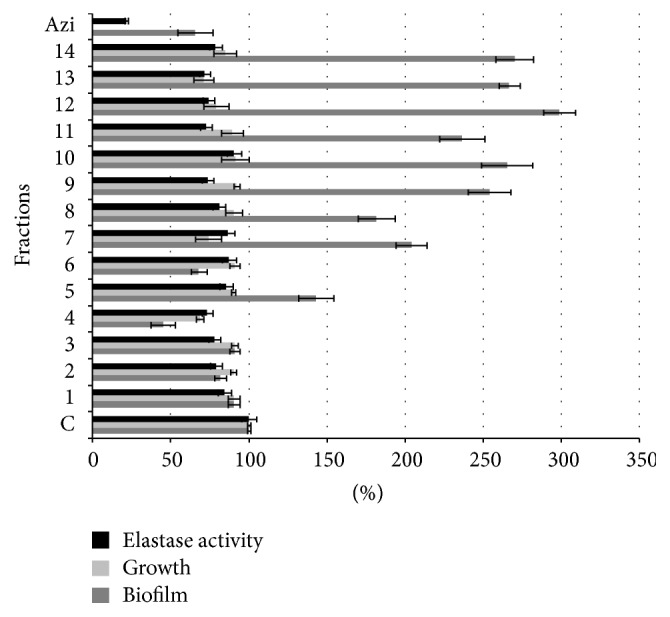
Effects of fractions 1–14 from* Merremia dissecta* on* Pseudomonas aeruginosa* growth, biofilm production, and elastase activity. Azi: azithromycin, C:* Pseudomonas aeruginosa* ATCC 27853, and 1–14: fractions of* M. dissecta* diethyl ether extract. The error bars indicate standard deviation (*n* = 8). All experiments showed significant differences with the control (*P* < 0.05).

DEL fractionation yielded fourteen fractions of increasing polarity, and their comparative effects on* P. aeruginosa* growth, biofilm production, and *β*-elastase activity (%) were investigated here. As shown in Figure 1, fractions 1–4 and 6 inhibit biofilm formation; particularly fraction 4 eluted with petroleum ether-ethyl acetate 95 : 5 displayed the highest* P. aeruginosa* biofilm inhibition (55%). Growth and elastolytic activity were inhibited by 31% and 27%, respectively. Coherently, the specific biofilm produced, that is, the amount of biofilm that each bacterium forms [10] calculated as the relation between the biofilm developed (OD_540 nm_) and bacterial growth (OD_600 nm_), was lower than control (0.6537).

It is remarkable that fractions 5 and 7–14 showed a strong increase of biofilm formation after 24 h of incubation, although the* P. aeruginosa* growth was not notably inhibited as most stressors would. This composition-specific behavior is consistent with previous publications that demonstrated that small chemical changes exert opposite effects (inhibition-stimulation) [8, 10]. According to our previous results [7, 32], this important stimulant effect could be exploited to obtain a higher biofilm-biomass of polycyclic aromatic hydrocarbon degrading* Pseudomonas* that would improve strategies for optimizing the carcinogenic substances bioremediation process.

In addition, the elastolytic activity of* P. aeruginosa* was significantly reduced by all fractions compared to control (13%–28%, *P* < 0.05).

### 3.6. Antiquorum Sensing Mode of Action of* M. dissecta* Fractions

**Figure 2:**
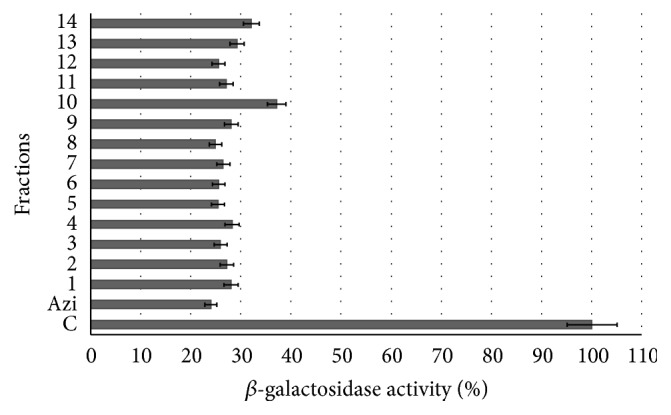
Quantification of* N*-acyl homoserine lactones signals by *β*-galactosidase activity. Azi: azithromycin supernatant, C:* P. aeruginosa* ATCC 27853 supernatant, and 1–14: fractions of* M. dissecta* diethyl ether extract (supernatants). The error bars indicate standard deviation (*n* = 8). All experiments showed significant differences with the control supernatant (*P* < 0.05).

All assayed fractions produced a notable decrease of the* P. aeruginosa* autoinducer biosynthesis (63–75%). Particularly, fraction 4 showed an AHL activity reduction of 72% (Figure 2). The observed effects are correlated with the biofilm formation and elastase activity decrease except for fractions 5 and 7–14 that produced more biofilm than control (Figure 1). This lack of correspondence could be due to another mechanism involved in biofilm formation.

It is very important to find plant natural products that decrease AHL production, since AHLs are signals utilized by Gram negative pathogenic bacteria to enable host colonization through AHL-mediated inhibition of induced inflammation via innate immune receptor mechanisms [33]. These signalling molecules inhibit lymphocyte proliferation and tumour necrosis factor-*α* production. They also decrease interleukin-12 production in lipopolysaccharide-stimulated macrophages [34].

### 3.7. Chemical Composition of the Bioactive Fraction 4

**Figure 3:**
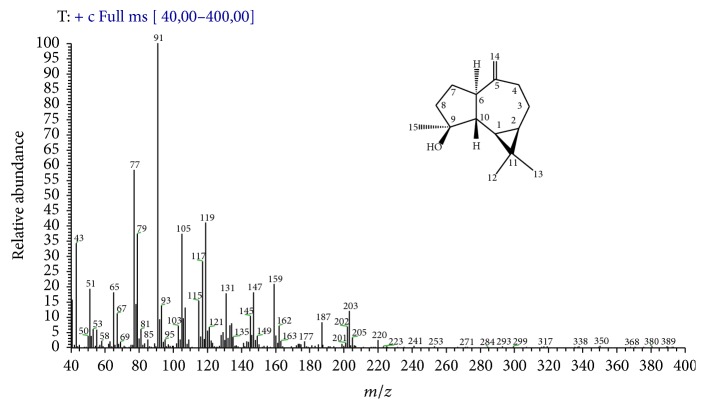
EIMS profile of fraction 4 (+ spathulenol) and the assignments of the main mass fragmentation peaks. MS [*m*/*z*]: 220 [M^∙+^], 205 [M–CH_3_]^+^, 203 [M–OH]^+^, 202 [M–H_2_O]^∙+^, 187 [M–CH_3_–H_2_O]^+^, 177 [M–CH_3_–C=O]^+^ or [M–CH_3_–CH_2_=CH_2_]^+^, 163 [C_12_H_19_]^+^, 159 [C_12_H_15_]^+^, 147 [C_11_H_15_]^+^, 145 [C_11_H_13_]^+^, 119 [C_9_H_11_]^+^, 117 [C_9_H_9_]^+^, 105 [C_8_H_9_]^+^, 91 [C_7_H_7_]^+^ (100%), 77 [C_6_H_5_]^+^, 67 [C_5_H_7_]^+^, 65 [C_5_H_5_]^+^, 51 [C_4_H_3_]^+^, and 43 [C_3_H_7_]^+^.

Spathulenol was found in fraction 4 by GC-EIMS and NMR analysis. Indeed, the NMR data of fraction 4 were identical to those previously reported for (+) spathulenol [35], and its EIMS profiling and the assignments of fragment ions are shown here (Figure 3). Hence, these results lead us to suppose that the antipathogenic effects exerted by fraction 4 can be attributed to the occurrence of (+) spathulenol.

In addition, the antibacterial and antipathogenic properties of stereoisomer (−)* ent*-spathulenol isolated from* Porella* species were previously demonstrated by Gilabert et al. [8]. However, it is important to note that (+) spathulenol, isolated from leaves of the vascular plant* M. dissecta*, showed a better anti-*β*-galactosidase activity than (−) isomer found in liverworts.

## 4. Conclusions

The bioguided study of the subtropical Convolvulaceae species assayed provides evidence of the occurrence of antipathogenic natural products active against Gram positive and negative bacteria. Particularly, eukaryotic metabolites such as (+) spathulenol from the ethnomedicinal plant* M. dissecta* are able to interfere with the* Pseudomonas aeruginosa* QS system by a strong decrease of the AHL biosynthesis (72%) which attenuates virulence factor expression.

The biofilm matrix is a key therapeutic target, and our findings suggest that the signalling pathway interruption by natural products of* M. dissecta* could be exploited as a good strategy for the development of new and effective antipathogenic drugs.

## Figures and Tables

**Table 1:** Yields, UV absorptions, and antibacterial and antibiofilm activities of Convolvulaceae extracts.

Plants	Extract	Yield (%)	UV nm (Abs)	*P. aeruginosa* ATCC 27853				*S. aureus *ATCC 6538 P			
				OD_600_ nm	Growth	OD_540_ nm	Biofilm	OD_560_ nm	Growth	OD_540_ nm	Biofilm
				0.887 ± 0.08	100%	0.390 ± 0.05	100%	1.516 ± 0.02	100%	0.420 ± 0.08	100%
*I. cairica *	DEF	0.35	298 (0.734)	0.168 ± 0.082	19%	0.246 ± 0.001	63%	1.367 ± 0.071	90%	0.113 ± 0.05	27%
	DEL	0.51	325 (1.105)	0.165 ± 0.003	19%	0.246 ± 0.001	63%	1.493 ± 0.033	98%	0.097 ± 0.001	23%
			351 (0.933)								
	MEF	2.55	302 (1.697)	1.078 ± 0.075	122%	0.144 ± 0.014	37%	1.136 ± 0.141	75%	0.004 ± 0.001	1%
	MEL	2.88	304 (1.200)	1.212 ± 0.084	137%	0.101 ± 0.020	26%	1.311 ± 0.098	87%	0.021 ± 0.025	5%

*I. nil*	DEF	0.26	304 (1.181)	0.095 ± 0.045	11%	0.374 ± 0.05	96%	1.310 ± 0.084	86%	0.076 ± 0.021	18%
			380 (0.729)								
	DEL	0.46	322 (0.904)	0.301 ± 0.053	34%	0.400 ± 0.189	102%	1.352 ± 0.048	89%	0.067 ± 0.042	16%
			343 (0.861)								
	MEF	1.43	298 (1.005)	0.864 ± 0.081	97%	0.183 ± 0.001	47%	1.406 ± 0.131	93%	0.021 ± 0.013	5%
	MEL	3.11	304 (0.845)	1.042 ± 0.065	118%	0.226 ± 0.018	58%	1.297 ± 0.099	86%	0.063 ± 0.02	15%

*I. purpurea*	DEF	0.43	300 (1.013)	0.154 ± 0.007	17%	0.261 ± 0.015	67%	1.314 ± 0.066	86%	0.063 ± 0.01	15%
	DEL	1.45	263 (0.136)	0.114 ± 0.053	13%	0.420 ± 0.05	108%	1.516 ± 0.108	100%	0.122 ± 0.05	29%
	MEF	3.05	302 (1.853)	1.123 ± 0.081	127%	0.148 ± 0.021	38%	1.547 ± 0.105	102%	0.055 ± 0.01	13%
			351 (1.450)								
	MEL	2.13	318 (2.171)	1.115 ± 0.075	126%	0.214 ± 0.032	55%	1.441 ± 0.076	95%	0.067 ± 0.02	16%
			351 (1.450)								

*M. dissecta*	DEF	0.06	305 (0.76)	0.141 ± 0.017	16%	0.296 ± 0.04	76%	1.365 ± 0.120	90%	0.038 ± 0.01	9%
	DEL	0.90	322 (1.368)	0.129 ± 0.013	15%	0. 078 ± 0.03	20%	1.243 ± 0.060	82%	0.063 ± 0.03	15%
			351 (1.184)								
	MEF	1.57	351 (1.390)	0.297 ± 0.014	34%	0.136 ± 0.000	35%	1.330 ± 0.123	88%	0.000 ± 0.001	0%
			367 (1.517)								
	MEL	1.94	330 (2.447)	0.793 ± 0.061	89%	0.257 ± 0.03	66%	1.434 ± 0.095	95%	0.034 ± 0.02	8%
			351 (1.799)								

*I.*: *Ipomoea. M.*: *Merremia*. DEF: diethyl ether extracts from flowers. DEL: diethyl ether extracts from leaves.

MEF: methanol extracts from flowers. MEL: methanol extracts from leaves.

All experiments showed significant differences with the controls (*n* = 8, *P* < 0.05).

**Table 2:** Volatile metabolites of *M. dissecta* diethyl ether extract.

RT^a^ (min)	RI^b^	Compounds	Structure	MW^c^	Main mass fragmentation peaks (*m*/*z*)	%
8.79	1036	Benzyl alcohol	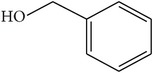	108	108 (M^+•^), 107, 91, 79 (100%), 63 and 51	11.17

14.49	1150	Benzoic acid	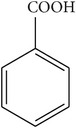	122	122 (M^+•^), 105 (100%), 77 and 51	3.36

20.80	1377	*δ*-Elemene	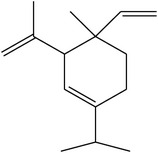	204	204 (M^+•^), 189, 175, 161, 148, 136, 121 (100%), 105, 93, 77, 67 and 53	2.65

23.16	1398	*β*-Elemene	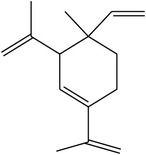	204	189, 147, 107, 81 (100%) and 68	4.70

24.45	1494	*β*-Caryophyllene	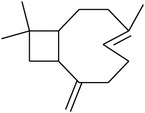	204	204 (M^+•^), 189, 161, 133, 105 and 93 (100%)	13.47

27.6	1515	Germacrene D	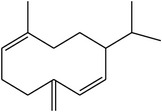	204	204 (M^+•^), 161 (100%), 105, 79 and 55	25.56

31.05	1536	Spathulenol	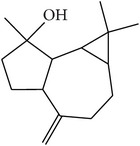	220	220 (M^+•^), 205, 187, 159, 131, 119, 91 (100%), 79 and 55	6.27

38.9		Not identified				5.83

45.6	1869	Pentadecanoic acid	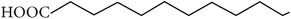	242	242 (M^+•^), 199, 143, 129, 115, 87, 73 (100%), and 60	13.52

45.65	2167	Stearic acid	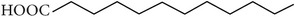	284	284 (M^+•^), 265, 241, 185, 143, 129, 115, 97, 83, 73 (94%) and 57	3.76

^a^RT: retention time. ^b^RI: Kovats retention indexes. ^c^MW: molecular weight.
